# Osseous Sarcoidosis: Three Typical Presentations of an Atypical Disease

**DOI:** 10.7759/cureus.84579

**Published:** 2025-05-21

**Authors:** Lauren C Ray, Frehiywot Ayele

**Affiliations:** 1 Internal Medicine, Emory University School of Medicine, Atlanta, USA; 2 Rheumatology, Emory University School of Medicine, Atlanta, USA

**Keywords:** multiorgan sarcoidosis, osseous sarcoidosis, sarcoidosis, sarcoidosis progression, sarcoidosis symptoms

## Abstract

Sarcoidosis is a systemic inflammatory disease characterized by the formation of non-necrotizing granulomas in affected tissues. While it most commonly involves the lungs and intrathoracic lymph nodes, it can affect virtually any organ system. Osseous sarcoidosis is a less common manifestation, and its prevalence may be underrepresented, as many patients remain asymptomatic. When symptoms do occur, they may include bone pain, reduced exercise capacity, or localized swelling. The spine is the most frequently involved site. In this case series, we present three distinct patients with osseous sarcoidosis: Patient A was diagnosed with sarcoidosis at age 31, initially presenting with pulmonary and sinonasal involvement. Years later, he developed dactylitis in his right third digit. A hand X-ray revealed osseous sarcoidosis in multiple fingers, despite symptoms being limited to a single digit. He was treated with methotrexate and infliximab, with significant improvement. Patient B was diagnosed at age 40 with predominant neurological involvement. He later developed back pain, and imaging revealed lesions throughout the lumbar and sacral spine. A biopsy confirmed osseous sarcoidosis. Initial treatment included cyclophosphamide, and he is now maintained on azathioprine and adalimumab, which he tolerates well. Patient C was diagnosed at age 55. At 68, she developed neck and back pain. Imaging showed faint sclerotic lesions throughout the thoracic spine, raising concern for either metastatic disease or osseous sarcoidosis. Biopsy confirmed sarcoidosis. Despite treatment with infliximab, azathioprine, and low-dose prednisone, she continues to experience neck pain, which is managed with gabapentin. Sarcoidosis severity and clinical progression vary widely between individuals and can be unpredictable. Notably, all three patients in this series had initial presentations that did not involve bone, with osseous sarcoidosis being identified years after their initial diagnoses. At the time of osseous involvement, all had multi-organ disease. Interestingly, each case also revealed asymptomatic osseous lesions alongside symptomatic sites, suggesting that subclinical skeletal involvement may coexist even in patients presenting with localized symptoms. Early identification of osseous sarcoidosis may be valuable in recognizing patients at risk for more severe or multi-organ disease.

## Introduction

Sarcoidosis is a multi-organ disease characterized by the infiltration of non-necrotizing granulomas, most commonly affecting the lungs and intrathoracic lymph nodes [1]. Diagnosis requires a compatible clinical presentation, histopathologic evidence of non-necrotizing granulomatous inflammation, and the exclusion of alternative diagnoses [2]. In the United States, the prevalence of sarcoidosis is approximately 60 per 100,000 adults, although this varies by sex and race, with the highest incidence and prevalence observed among women and African Americans [3].

Osseous sarcoidosis is a less common manifestation, reported in approximately 0.5-30% of patients with sarcoidosis [1,4,5]. However, these estimates may underrepresent the true prevalence, as many patients with osseous involvement are asymptomatic [6]. When present, symptoms can include bone pain, reduced exercise capacity, or swelling. The spine is the most frequently affected site [5,6]. Most patients with osseous sarcoidosis also exhibit other systemic signs of the disease, such as lymphadenopathy or parenchymal lung involvement [5,7].

Initial diagnosis typically relies on imaging. For the axial skeleton, PET-CT and MRI are most useful, while plain radiographs may be used for the appendicular skeleton [6]. Notably, about 50% of osseous sarcoidosis cases are asymptomatic and incidentally discovered through imaging performed for unrelated reasons. Radiologic findings may include sclerotic, osteolytic, or cystic lesions or cortical abnormalities [8]. A significant diagnostic challenge is that these lesions often cannot be reliably distinguished from osseous metastases - and less commonly, from osteoporosis or Paget’s disease - based on imaging alone. Therefore, biopsy is frequently necessary for definitive diagnosis [9].

Recognition of osseous sarcoidosis is clinically important, as it often reflects underlying multi-organ involvement. Progression or emergence of osseous disease may indicate a higher susceptibility to the involvement of other organs, such as the liver or spleen [6]. Accordingly, patients with osseous sarcoidosis may benefit from more aggressive treatment strategies, not solely due to bone involvement but because of the broader trajectory of their systemic disease.

In this report, we present three unique cases of osseous sarcoidosis, detailing the diagnostic process, treatment approaches, and patient responses.

## Case presentation

Patient A

Patient A is a 41-year-old male who was diagnosed with sarcoidosis at age 31 after experiencing weight loss, cough, nausea, and vomiting. Chest CT revealed hilar adenopathy, and bronchoscopy with biopsy showed non-necrotizing granulomas. He was initially treated with prednisone monotherapy at 20 mg daily. Due to well-controlled symptoms, prednisone was discontinued after a few years, and no steroid-sparing agents were initiated at that time.

Approximately five years after diagnosis, he developed hoarseness, and otolaryngologic evaluation revealed a hard palate perforation and a large nasal septal perforation. These findings were suspected to represent sinonasal involvement of sarcoidosis. He was started on methotrexate 15 mg weekly and a prednisone taper beginning with 10 mg daily for 30 days, followed by 5 mg daily. Methotrexate was well tolerated, and the dose was gradually increased to 20 mg weekly and then 25 mg weekly.

**Figure 1:**
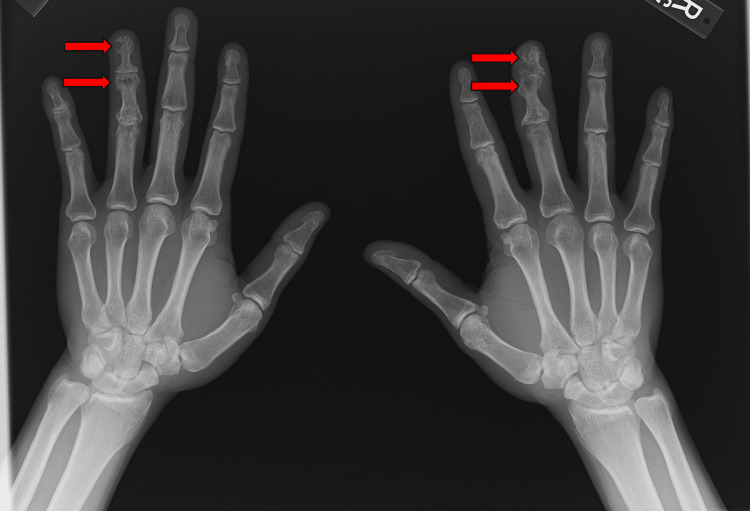
Bilateral hand X-ray showing osseous sarcoidosis affecting the right third digit and left fourth digit

Follow-up imaging showed worsening pulmonary disease consistent with organizing pneumonia. On examination, he had dactylitis of the right third digit. A hand X-ray revealed increased lucency and trabecular thinning of the right third distal and middle phalanges, as well as trabecular changes in the left fourth distal and middle phalanges (Figure 1). These findings were consistent with osseous sarcoidosis. At the time of the X-ray, his angiotensin-converting enzyme level was elevated at 136 U/L (reference range: 16-85 U/L).

He was started on infliximab infusions at 5 mg per kg every six weeks, his prednisone was increased to 10 mg daily, and he continued methotrexate at 25 mg weekly. Both his dactylitis and pulmonary symptoms improved significantly and have remained stable since.

Patient B

Patient B is a 47-year-old male with sarcoidosis involving the brain, lymph nodes, heart, liver, and bones. He was diagnosed with sarcoidosis at age 40, initially presenting with three months of headache, gait disturbance, horizontal diplopia, and dizziness. Imaging revealed leptomeningitis of the posterior fossa, and cerebrospinal fluid analysis showed low-grade lymphocytic pleocytosis, elevated protein, and hypoglycorrhachia. Chest CT demonstrated mediastinal and axillary lymphadenopathy, while abdominal and pelvic CT revealed enlarged cardiophrenic, mesenteric, retroperitoneal, and bilateral external iliac lymph nodes. Biopsy of a right external iliac lymph node confirmed non-necrotizing granulomas.

His initial maintenance therapy included prednisone 60 mg daily; however, treatment was complicated by significant weight gain. He was then started on infliximab 7.5 mg/kg every four weeks and methotrexate 15 mg weekly as steroid-sparing agents, and prednisone was tapered. Although his symptoms initially improved, he later developed worsening headaches, short-term memory loss, and imbalance. Brain MRI showed severe diffuse leptomeningeal enhancement. Antibody testing revealed he had developed antibodies to infliximab, prompting discontinuation of both infliximab and methotrexate. He was subsequently started on monthly cyclophosphamide infusions at 1,000 mg. His symptoms, particularly headache, improved, and follow-up MRIs demonstrated near-complete resolution of intracranial enhancement.

**Figure 2:**
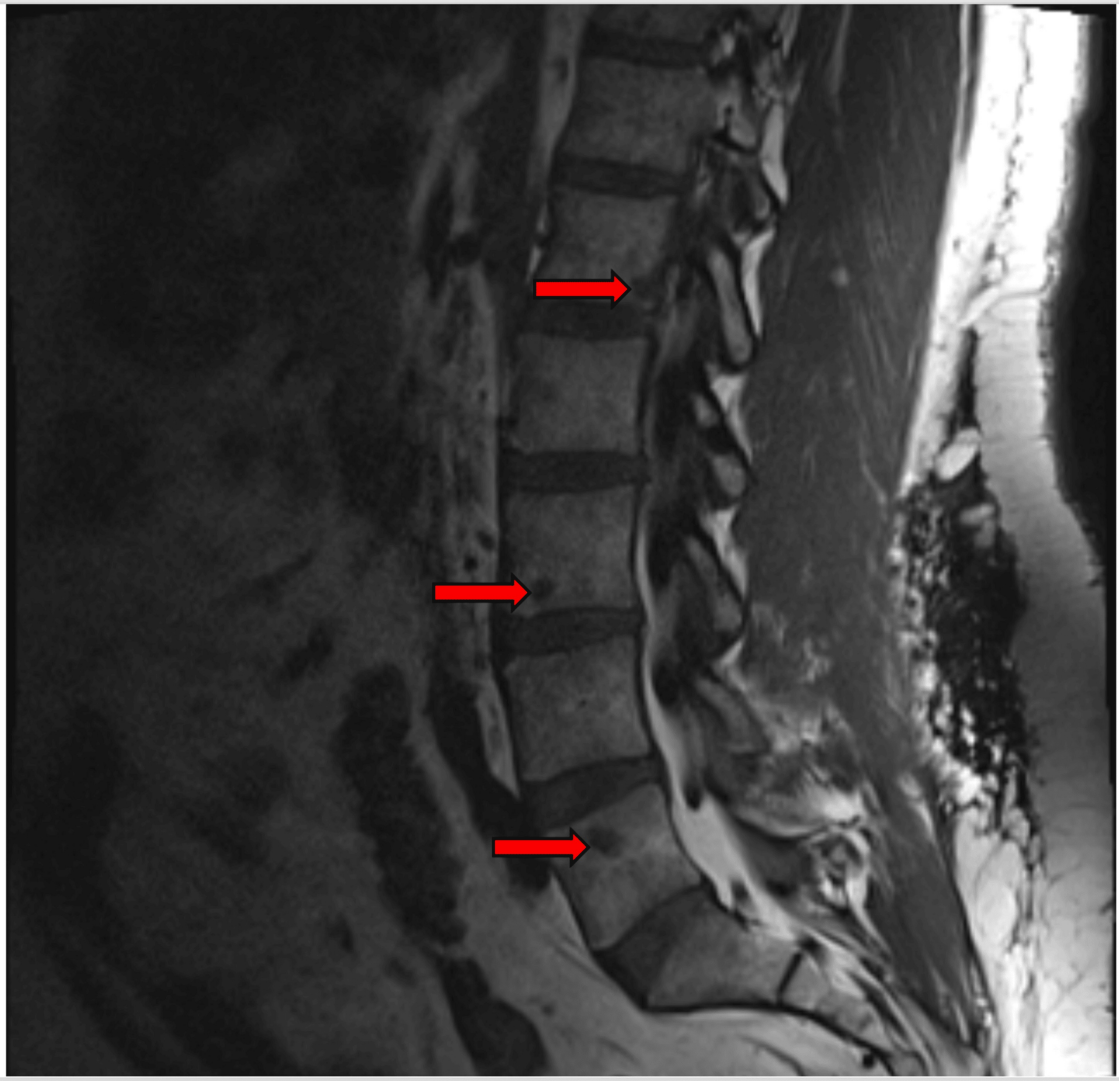
MRI of the lumbar spine showing multifocal hypointense lesions consistent with osseous sarcoidosis Three lesions are indicated by red arrows.

While receiving cyclophosphamide, he developed non-radiating lower back pain that worsened with ambulation. Lumbar spine MRI revealed new multifocal enhancing T1 hypointense and T2 hyperintense lesions throughout the lumbar and sacral spine and pelvis, which were not present on imaging from two years prior (Figure 2). The radiologic differential included osseous sarcoidosis versus metastatic disease. PET-CT demonstrated hypermetabolic adenopathy in the neck, chest, abdomen, and pelvis, with focal involvement of the spleen and bones. Biopsy of the right iliac bone confirmed non-necrotizing granulomas without evidence of malignancy, consistent with osseous sarcoidosis.

After six months of cyclophosphamide therapy, he was transitioned to adalimumab 40 mg every two weeks, initially combined with methotrexate 10 mg weekly to prevent antibody formation. At present, he remains on azathioprine 50 mg daily and adalimumab 40 mg every two weeks, both of which he tolerates well.

Patient C

Patient C is a 69-year-old female who was diagnosed with sarcoidosis at age 55 after presenting with fatigue, shortness of breath, cough, wheezing, night sweats, and difficulty balancing. Bronchoscopy with transbronchial biopsy revealed non-necrotizing granulomas. She was initially treated with high-dose prednisone (exact dose unknown) but developed significant weight gain and insulin-dependent diabetes mellitus. Methotrexate 15 mg weekly was then started while prednisone was tapered to 10 mg daily; however, she discontinued methotrexate due to side effects. Her treatment was switched to azathioprine 150 mg daily, and prednisone was further reduced to 2.5 mg daily.

**Figure 3:**
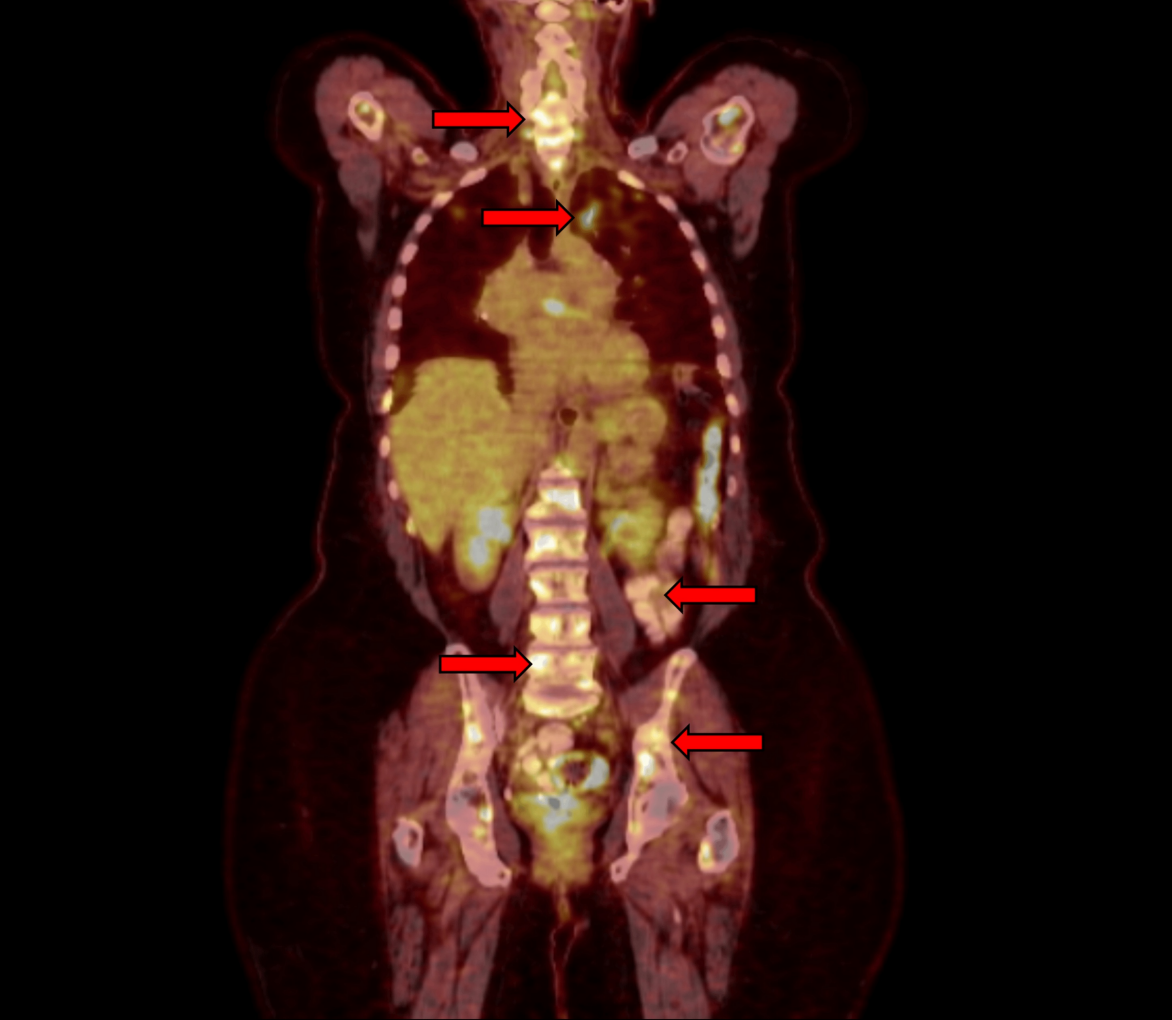
PET-CT scan showing FDG-avid lesions in the bones consistent with osseous sarcoidosis, along with non-osseous FDG-avid lesions in the neck, chest, abdomen, and pelvis FDG, fluorodeoxyglucose

At age 68, she began experiencing neck and back pain. A CT scan revealed faint sclerotic lesions throughout the thoracic spine, raising concern for metastatic disease versus osseous sarcoidosis. PET-CT showed multiple diffusely fluorodeoxyglucose-avid lesions in the axial and appendicular skeleton as well as in the abdomen (Figure 3). MRI revealed innumerable enhancing lesions suggestive of sarcoidosis or metastasis. Bone biopsy confirmed non-necrotizing granulomas without malignancy. Around the onset of her neck and back pain, she also developed headaches. Brain MRI demonstrated leptomeningeal enhancement in the left frontal lobe, consistent with neurosarcoidosis.

Her treatment was escalated to include infliximab infusions at 5 mg/kg every six weeks, while prednisone was maintained at 2.5 mg daily and azathioprine was reduced to 50 mg daily. Despite this regimen, her leptomeningeal disease remained stable. Although improved compared to prior assessments, she continued to experience neck pain, back pain, and headaches, which she managed with gabapentin 300 mg three times daily.

## Discussion

Osseous sarcoidosis is a relatively uncommon manifestation of sarcoidosis, although its true prevalence remains uncertain. In this report, we present three cases illustrating different clinical presentations of osseous sarcoidosis: dactylitis, back pain, and neck pain (Table 1). Each patient initially presented with sarcoidosis that did not involve the bones, with osseous disease developing years later. Additionally, all three patients had multi-organ involvement at the time their osseous disease was identified, consistent with previous studies linking osseous sarcoidosis to multisystem disease [5-7]. Of these cases, two required bone biopsy for confirmation, as imaging alone could not reliably differentiate osseous sarcoidosis from metastatic or other bone diseases - a challenge well documented in the literature [9-12].

**Table 1:** Initial presentation, diagnostic imaging, treatment, and non-osseous manifestations of the three patients with osseous sarcoidosis

Patient	Initial presentation	Imaging	Bone biopsy proven?	Treatment	Non-osseous sarcoidosis manifestations
A	Dactylitis	X-ray	No	Infliximab 5 mg/kg every six weeks and methotrexate 25 mg weekly	Sinonasal, mediastinal lymph nodes, pulmonary
B	Lower back pain	PET-CT, MRI	Yes	Initially, cyclophosphamide 1,000 mg monthly, then azathioprine 50 mg daily, and adalimumab 40 mg every two weeks	CNS, diffuse lymph nodes, heart, liver, spleen
C	Neck and back pain	PET-CT, MRI	Yes	Infliximab 5 mg/kg every six weeks, prednisone 2.5 mg daily, and azathioprine 50 mg daily	Mediastinal lymph nodes, pulmonary, CNS

Because much of the existing data on osseous sarcoidosis comes from case reports and case series, there is currently no established consensus on its medical management. A retrospective case-control study involving 48 patients with osseous sarcoidosis found that glucocorticoids are a commonly used and effective treatment, particularly when combined with methotrexate or hydroxychloroquine [7]. There is also evidence supporting the use of tumor necrosis factor-alpha inhibitors, especially in patients with systemic involvement [5,7].

In our three cases, treatment decisions were not driven by the presence of osseous sarcoidosis alone. In the first patient, infliximab was initiated due to progression of pulmonary disease, although this also led to improvement in dactylitis symptoms. Similarly, in the second and third patients, the therapeutic approach was not altered specifically because of the osseous findings.

Interestingly, all three patients had asymptomatic osseous lesions in addition to symptomatic ones. For example, the first patient showed osseous involvement in the left hand, despite only having dactylitis in the right third digit. The second and third patients both had diffuse lesions, including in the pelvis, despite experiencing pain localized only to the spine. This may suggest that even in symptomatic cases, osseous sarcoidosis can also be present in other skeletal regions without causing symptoms.

Another important consideration is that spinal pain is not specific to osseous sarcoidosis. A systematic review of the general population found that the global mean lifetime prevalence of low back pain is approximately 38.9% [13]. It remains possible that the sarcoid lesions were incidental and unrelated to the patients’ spinal pain. However, this case series cannot establish causality due to multiple confounding factors and the absence of a control group. Given the small sample size, these findings should not be generalized.

Further research involving larger cohorts is needed to better understand why only certain osseous sarcoid lesions cause pain, whether there is value in treating asymptomatic osseous lesions, and what the optimal treatment strategies are when intervention is warranted.

Overall, these three cases provide insight into the varied presentations, diagnostic approaches, and treatment considerations in osseous sarcoidosis. Due to its low prevalence, much remains to be learned, particularly regarding its implications for disease progression and optimal therapeutic management.

## Conclusions

Sarcoidosis severity varies significantly between individuals, and its clinical course can be unpredictable. Osseous sarcoidosis is a relatively rare manifestation and may present asymptomatically or with symptoms such as pain, swelling, or decreased exercise tolerance. Early identification of osseous involvement may help raise clinical suspicion for patients at higher risk of developing severe or multi-organ disease.
